# The Prototypes of Tobacco Users Scale (POTUS) for Cigarette Smoking and E-Cigarette Use: Development and Validation

**DOI:** 10.3390/ijerph17176081

**Published:** 2020-08-21

**Authors:** Eboneé N. Butler, Marissa G. Hall, May S. Chen, Jessica K. Pepper, Hart Blanton, Noel T. Brewer

**Affiliations:** 1 Department of Epidemiology, Gillings School of Global Public Health, University of North Carolina, Chapel Hill, NC 27599 USA; ebonee@alumni.unc.edu; 2 Department of Health Behavior, Gillings School of Global Public Health, University of North Carolina, Chapel Hill, NC 27599, USA; mghall@unc.edu (M.G.H.); maychen@email.unc.edu (M.S.C.); 3 Lineberger Comprehensive Cancer Center, University of North Carolina, Chapel Hill, NC 27599, USA; 4 RTI International, Research Triangle Park, NC 27709, USA; jpepper@rti.org; 5 Department of Communication, Texas A&M University, College Station, TX 77843, USA; hblanton@tamu.edu

**Keywords:** prototypes, scale development, scale validation, tobacco, cigarettes, e-cigarettes

## Abstract

Endorsing prototypes of cigarette smokers predicts cigarette smoking, but less is known about prototypes of users of other tobacco products. Our study sought to establish the reliability and validity of a measure of prototypes of smokers and e-cigarette users. Participants were from a national survey of smokers and non-smokers (*n* = 1414), a randomized clinical trial (RCT) of adult smokers (*n* = 2149), and adolescent children of adults in the trial (*n* = 112). The Prototypes of Tobacco Users Scale (POTUS) has four positive adjectives (cool, sexy, smart, and healthy) and four negative adjectives (disgusting, unattractive, immature, and inconsiderate) describing cigarette smokers and e-cigarette users. Confirmatory factor analyses identified a two-factor solution. The POTUS demonstrated strong internal consistency reliability in all three samples (median α = 0.85) and good test–retest reliability among adults in the RCT (median r = 0.61, 1–4 weeks follow-up). In the RCT, smokers more often agreed with negative prototypes for smokers than for e-cigarette users (mean = 2.03 vs. 1.67, *p* < 0.05); negative prototypes at baseline were also associated with more forgoing of cigarettes and making a quit attempt at the end of the trial (Week 4 follow-up). The POTUS may be useful to public health researchers seeking to design interventions that reduce tobacco initiation or cessation through the manipulation of tobacco user prototypes.

## 1. Introduction

Social experiences play a meaningful role in shaping health behaviors early in life, from late childhood through young adulthood [1,2]. During these developmental stages, new and unfamiliar situations present opportunities for youth and young adults to make consequential decisions about risky behaviors such as tobacco use. The Prototype/Willingness (P/W) model posits that two separate processes motivate people to engage in risk behaviors [3]. One process relies on reasoned action. Rational thoughts lead to intentions to act that lead to risky (or protective) behavior. A second process relies on responses to social pressure. The social images of those engaged in a behavior—known as prototypes—increase willingness to act, which increases the likelihood of engaging in the behavior when the opportunity presents itself [3]. The more passive social process, which relies on prototypes, may be especially worrisome for adolescents and others who do not have explicit and relevant health-related goals to engage in a risk behavior [4].

Administration of social prototype measures in research studies might prove particularly informative to public health experts seeking to develop effective anti-tobacco interventions. By measuring perceptions of the “typical” person engaged in smoking or other tobacco use, researchers can gain a better understanding of the underlying images that influence initiation, continued use, and attempts at cessation [5]. Indeed, prototype endorsements have been shown to predict cigarette smoking in longitudinal studies of children [6], adolescents [7], and young adults [8]. While social prototypes are commonly believed to have the greatest influence during childhood or adolescence [2,3,9], smoker prototypes have also proved useful in understanding motivations among adult smokers and have been used to predict success at quit attempts [10,11]. Nevertheless, conceptualizations of smoker prototypes have varied across studies (Table 1). A standard, valid, and reliable measure of tobacco user prototypes would aid public health researchers in characterizing contemporary smoker prototypes to examine their impact on tobacco use.

Electronic cigarettes (e-cigarettes) and other electronic vapor products have gained in popularity in recent years [18,19,20,21]. Between 2011 and 2016, the percentage of U.S. youth reporting past 30 days use of e-cigarettes rose from 2% to 11% among high school students and from 1% to 4% among middle school students. These figures represent nearly 4 million youth e-cigarette users in 2016 [21]. In 2014, 13% of U.S. adults had ever tried an e-cigarette, and 4% currently used them [19], compared to 3% and 1%, respectively, in 2010 [20]. E-cigarettes are likely to be safer overall than cigarettes [22]; however, vaping carries risks that are increasingly well documented and worrisome [23,24]. Many e-cigarettes contain nicotine, a chemical that can lead to addiction [22] and harm adolescent brain development [25]. Some evidence suggests that e-cigarette use may also lead to cigarette smoking among adolescents and adults who would not otherwise smoke [26,27,28,29].

With the growing uptake of e-cigarettes and its potential link to smoking initiation, it is important to have measures that can predict e-cigarette use. Critical to any such efforts will be the development of a new class of social prototype measures designed to capture the attributes and traits associated with the “typical” cigarette or e-cigarette user. At present, the field lacks a robust measure for tobacco user prototypes that is suitable for cigarette smoking and e-cigarette use. To address this gap, we describe the development of the Prototypes of Tobacco Users Scale (POTUS). We evaluate whether established prototypes of smokers accurately reflect positive and negative perceptions of both cigarette and e-cigarette users, and we examine relationships between tobacco user prototypes and attitudes, behaviors, and beliefs linked to smoking and e-cigarette use in adolescent and adult samples.

## 2. Materials and Methods

### 2.1. Participants and Procedures

The three studies described in this article were approved by the Institutional Review Board at the University of North Carolina at Chapel Hill (adult online study # 12-2063, adult RCT #13-2861, adolescent study #14-2011). Study participants provided written consent for all three studies.

#### 2.1.1. Adult Online Study

In 2014, we recruited a national convenience sample of 1414 U.S. adults through Amazon Mechanical Turk (MTurk), an online recruitment tool that has been widely used in social science research and is recognized for yielding valid and reliable survey data for some groups [30,31,32]. To be eligible for inclusion, study participants had to be age 18 or older, speak English, and live in the United States. Participants received $3 as compensation for completing an online survey. Participant characteristics appear in Supplementary Table S1.

#### 2.1.2. Adult Randomized Clinical Trial (RCT)

In 2014 and 2015, we recruited 2149 U.S. adult smokers for a 4-week RCT in North Carolina and California, USA [27]. The trial aimed to assess the effects of pictorial cigarette pack warnings on quit attempts. Details of the study design, recruitment, and procedures appear elsewhere [33,34]. During the 4-week trial, participants visited the study site each week and completed a computer-based survey. At the baseline visit, smokers completed a survey (referred to as T1), inspected their cigarette packs after warnings were applied, and completed a second survey (T2); we refer to time points for data collected in subsequent weekly return visits as T3, T4, T5, and T6. Participants received an incentive of up to $185 in North Carolina and $200 in California, depending on the number of surveys completed.

#### 2.1.3. Adolescent Study

Finally, we recruited 112 adolescent children of adults (age range: 13–17 years) who participated in the RCT for an ancillary study of responses to pictorial warnings on the parents’ cigarette packs. A full description of the study design, recruitment, and procedures appears elsewhere [35]. We conducted phone interviews with the adolescents and provided a $40 incentive. The University of North Carolina Institutional Review Board approved all three studies.

### 2.2. Measures

#### 2.2.1. Prototypes

To develop the Prototypes of Tobacco Users Scale (POTUS), we relied on previous studies among adults and adolescents (Table 1) to identify a common set of adjectives used to describe prototypes of tobacco users. In a preliminary MTurk survey with 125 adults, we also solicited feedback on adjectives that would describe typical cigarette smokers and e-cigarette users. We examined the extent to which their open-ended responses overlapped with adjectives used in the extant literature and considered potential new adjectives relevant to e-cigarette user prototypes. Ultimately, we selected the most frequently observed adjectives reported in the preliminary survey that had also been previously described in the literature. Based on this evaluation, we identified 8 adjectives that were common across perceptions of smokers and e-cigarette users.

In the adult online study and adult RCT, the POTUS survey stem read, “How much do the following characteristics describe a typical cigarette smoker your age?” In the adolescent study, we adapted the stem for phone interviews by asking: “How much does the word [adjective] describe a typical cigarette smoker user your age?” The surveys included parallel items for e-cigarette users. The POTUS has 4 positive adjectives (cool, sexy, smart, and healthy) and 4 negative adjectives (disgusting, unattractive, immature, and inconsiderate); we refer to the two adjective subsets as POTUS+ and POTUS–, respectively. The 5-point response scale was coded as strongly disagree (1), somewhat disagree (2), neither agree nor disagree (3), somewhat agree (4), and strongly agree (5).

#### 2.2.2. Other Measures

The studies of adults assessed positive and negative attitudes toward smokers and e-cigarette users. In the adult online study, the Attitude +/− survey items read, “Think about only your positive opinions of typical cigarette smokers your age, ignoring any negative opinions.” and “Think about only your negative opinions of typical cigarette smokers your age, ignoring any positive opinions.” In the adult RCT, the Attitude +/− survey items read, “How positive are your positive opinions of cigarette smokers?” and “How negative are your negative opinions of cigarette smokers?”. The adult RCT surveys included the same items about e-cigarette users. The 4-point response scale ranged from not at all positive/negative (coded as 1) to extremely positive/negative (4). All three studies included a global attitude item that read, “Picture a typical cigarette smoker your age. Is your opinion of this person…”. The adult RCT included a parallel item for e-cigarette users. The 5-point response scale for both items ranged from “very negative” (coded as 1) to “very positive” (5).

For the adult online study and adult RCT, we defined current smokers as having smoked at least 100 cigarettes during their lifetime and currently smoking every day or some days. For the adolescent study, we defined current smokers as having smoked a cigarette within the past 30 days. We defined current e-cigarette users as having used an e-cigarette in the past 30 days (adult online and adolescent studies) or in the past 28 days (adult RCT).

To support analyses of convergent validity, surveys in the adult online study and adult RCT measured number of cigarettes smoked per day and quit intentions (adult online study: 1 item [36]; adult RCT: 3 items [37], α = 0.87; Supplementary Table S2). The adult RCT surveys assessed subjective norms about quitting smoking [38] (4 items, α = 0.83), worry about the consequences of smoking [39,40] (4 items, α = 0.82), positive attitudes related to the look of the cigarette pack [41] (i.e., positive pack attitudes) (3 items, α = 0.87), negative attitudes toward one’s cigarette pack (i.e., negative pack attitudes) (3 items, α = 0.88), thinking about harms of smoking to oneself [42] (3 items, α = 0.61), and negative consequences of smoking (4 items, α = 0.81). Three items that assessed different aspects of social interactions surrounding e-cigarette were used in the adult RCT [43]. Finally, we measured exposure to e-cigarette advertising with a single item in the adult RCT. To support predictive validity analyses, the adult RCT measured number of times forgoing a cigarette in the past week [44,45] and whether smokers had made a quit attempt in the past week (“During the last week, did you stop smoking for 1 day or longer because you were trying to quit smoking?”, coded as yes (1) or no (0)) [46].

### 2.3. Data Analysis

We conducted confirmatory factor analysis using data from all three samples and modeled the POTUS as having two subscales (POTUS+ and POTUS–), representing positive and negative prototypes of smokers or e-cigarette users; these models allowed the POTUS+ and POTUS– subscales to correlate. We analyzed the three samples separately in order to examine potential differences across the factor loadings. The models used maximum likelihood estimation to calculate standardized factor loadings and error terms. We allowed the error terms for “cool” and “sexy” to correlate based on the results of the model respecification process [47,48]. To assess model fit, we examined the root mean square error of approximation (RMSEA; acceptable fit defined as <0.08) [49] and the Bentler Comparative Fit Index (CFI; acceptable fit defined as >0.95) [50]. To examine internal consistency reliability, we calculated Cronbach’s alpha using data from all three studies. To examine test–retest reliability, we examined correlations of the same measures across the multiple weeks of the adult RCT. To identify differences in smoker and e-cigarette user prototypes, we compared POTUS subscale means using paired t-tests with data from the adult RCT.

To assess convergent validity, we examined correlations between the POTUS subscales and smoking status, smoking frequency, and smoking-related attitudes and beliefs in the adult online study and the adult RCT. Using data from the adult RCT, we examined correlations between e-cigarette POTUS+, e-cigarette POTUS–, e-cigarette use, social interactions about e-cigarette use, and exposure to advertising for e-cigarettes. To assess predictive validity, using data from the adult RCT, we examined the association between the smoker POTUS subscales at baseline and two behavioral outcomes at T6 (i.e., Week 4 follow-up): forgoing a cigarette and making a quit attempt. Predictive validity analyses controlled for trial arm; thus, we present standardized regression coefficients for continuous outcomes (i.e., forgoing) and odds ratios for dichotomous outcomes (i.e., quit attempt). Finally, we examined correlations between POTUS subscales and attitudes toward smokers or e-cigarette users as captured by the attitude+, attitude-, and attitude global measures in the adult online study and the adult RCT. Analyses used SAS version 9.4 (SAS Institute, Cary, NC, USA) and STATA version 13.1 (StataCorp, College Station, TX, USA), with two-tailed tests and a critical alpha of 0.05. We report only statistically significant correlation coefficients in text.

## 3. Results

### 3.1. Scale Psychometrics

Confirmatory factor analyses demonstrated good model fit for the smoker and e-cigarette POTUSs for each study sample (RMSEA ranged from 0.00 to 0.07, CFI ranged from 0.98 to 1.00, Supplementary Table S3). Internal consistency reliability was high for the smoker POTUS+ and – subscales in the adult online study (α = 0.80/0.85) and the adult RCT (α = 0.79/0.82) and somewhat lower in the adolescent study (α = 0.54/0.73). The pattern was similar for the e-cigarette user POTUS+ and –, with high reliability in the adult online study (α = 0.84/0.88) and adult RCT (α = 0.86/0.86) and lower reliability in the adolescent study (α = 0.65/0.72). Test–retest reliability was good for the smoker POTUS+ and – subscales in the adult RCT (median r = 0.61 across the 4 weeks, Supplementary Table S4). Test–retest reliability was moderate for the e-cigarette POTUS+ and – subscales between baseline and 4 weeks (r = 0.51, 0.46).

### 3.2. Smoker vs. E-Cigarette User Prototypes

**Figure 1:**
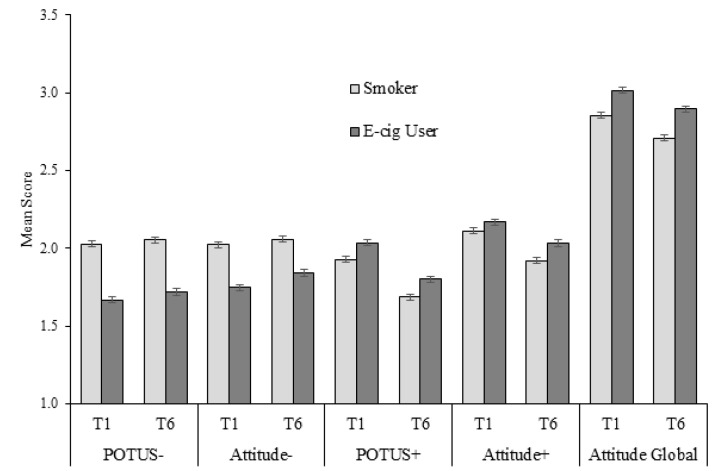
Prototypes of and attitudes toward smokers and e-cigarette users in the adult RCT. All comparisons of smokers and e-cigarette users *p* < 0.05. Error bars show standard errors.

In the adult RCT, negative prototypes were higher for smokers than for e-cigarette users (mean = 2.03 vs. 1.67, *p* < 0.05 at T1) and positive prototypes were lower for smokers than for e-cigarette users (mean = 1.93 vs. 2.03, *p* < 0.05 at T1) (Figure 1). We observed similar response patterns for the T1 and T6 time points. Similarly, the global attitude was more positive toward e-cigarette users than toward smokers (*p* < 0.05 at T1 and T6).

Adolescents also rated negative prototypes higher for smokers than for e-cigarette users (mean = 3.53 vs. 2.82, *p* < 0.05; data not shown), but did not rate positive prototypes differently for smokers and e-cigarette users (mean = 1.61 vs. 1.65, *p* > 0.05).

### 3.3. Validity

Compared to non-smokers, smokers had higher scores on the smoker POTUS+ and lower scores on the smoker POTUS– in the adult online study (r = 0.36/−0.42) (Table 2). Higher quit intentions were correlated with lower scores on the smoker POTUS+ and higher scores on the smoker POTUS– in the adult online study (r = −0.17/0.16) and adult RCT (r = −0.07/0.26).

The smoker POTUS+ was correlated with having more positive pack attitudes (r = 0.14 in the adult RCT) (Table 2). As expected, the smoker POTUS+ was negatively correlated with positive subjective norms, worry about the consequences of smoking, negative pack attitudes, and negative consequences of smoking (median r = −0.09). The smoker POTUS– was positively correlated with positive subjective norms about quitting smoking, worry about the consequences of smoking, negative pack attitudes, and negative consequences of smoking (median r = 0.36 for these measures in the adult RCT). In terms of predictive validity, the smoker POTUS– was also associated with more forgoing of cigarettes (β = 0.16, *p* < 0.05) and a higher likelihood of making a quit attempt (OR = 1.27, *p* < 0.05), both measured at the end of the 4-week trial. However, the smoker POTUS+ was not associated with either of these two outcomes at the end of the trial.

The e-cigarette POTUS+ was correlated with current e-cigarette use (r = 0.13) and frequency of use (r = 0.09) in the adult RCT (Table 2). The e-cigarette POTUS+ was also associated with talking to someone about e-cigarettes in the past month (r = 0.10) and recommending e-cigarettes to someone (r = 0.19) in the adult RCT. In the two adult samples, the attitude+, attitude–, and attitude global measures each demonstrated expected correlations with the POTUS+ and – subscales (Table 3).

## 4. Discussion

The tobacco control field lacks a standard measure of tobacco user prototypes. The Prototypes of Tobacco Users Scale (POTUS) fills this gap by identifying a psychometrically strong measure that can be used to evaluate the relationship between smoker or e-cigarette user prototypes and tobacco-related beliefs, attitudes, and behavior. Across three studies with adults and adolescents, confirmatory factor analyses pointed toward a two-factor scale with a positive prototype subscale (cool, sexy, smart, and healthy) and a negative prototype subscale (disgusting, unattractive, immature, and inconsiderate). In the two adult samples, the POTUS proved to be a reliable and valid measure for assessing perceptions about the “typical” smoker and e-cigarette user. The scale exhibited good test–retest reliability over a four-week period and strong internal consistency reliability, particularly among adults.

In support of the construct validity of the smoker POTUS, as expected, positive prototypes correlated with pro-smoking attitudes and beliefs, while negative prototypes correlated with anti-smoking attitudes and beliefs. For example, among adult smokers, positive smoker prototypes were associated with lower quit intentions, whereas negative smoker prototypes correlated with greater quit intentions. Importantly, the smoker POTUS was associated with smoking behavior; positive smoker prototypes correlated with being a smoker, and negative prototypes correlated with being a non-smoker. These findings build on prior research linking prototypes with smoking behavior among both adolescents [51,52] and adults [10]. In addition, we found that the smoker POTUS—at baseline in the adult RCT predicted cigarette forgoing and making a quit attempt at the end of the trial, supporting the predictive validity of this measure. However, we observed no association between the smoker POTUS+ and longitudinal outcomes. Thus, smokers who endorse negative prototypes may be most suitable for tobacco cessation interventions.

The magnitude of the correlations between negative smoker prototypes and the validity outcomes was generally higher than the correlations between positive smoker prototypes and the outcomes, supporting prior research that negative attitudes and beliefs have greater bearing on health behavior [53,54,55]. Thus, negative prototypes may play a critical role in tobacco prevention and smoking cessation efforts. Images that visually depict the negative consequences of smoking have been successful in aiding smoking cessation efforts through pictorial cigarette pack warnings and anti-smoking mass media campaigns [34,56]. Future interventions could benefit from increasing the degree to which smokers and non-smokers endorse negative smoker prototypes.

Data from the adult RCT also provided evidence supporting the construct validity of the e-cigarette POTUS. Positive e-cigarette user prototypes were associated with more conversations about e-cigarettes, recommending e-cigarettes to others, being an e-cigarette user, and frequency of e-cigarette use. As predicted, negative e-cigarette user prototypes correlated with being less likely to recommend e-cigarettes to others and not being an e-cigarette user. Adult study participants generally perceived e-cigarette users more positively than smokers, potentially due to the contemporary belief that e-cigarette use may be a safe alternative to smoking [57]. Though adolescents more often agreed with negative prototypes for smokers than for e-cigarette users, their rankings for positive prototypes did not differ between the two groups. Future research in both adult and adolescent populations should investigate the extent to which positive and negative e-cigarette prototypes might contribute to the growing popularity of e-cigarettes or highlight potential areas of intervention.

Strengths of the study include the theoretical and empirical basis for selecting the POTUS adjectives, the novel contribution of an e-cigarette user prototypes measure, and the use of three independent samples. However, due to the use of convenience samples, the generalizability of our findings to the U.S. population remains to be established. It is important to note that the interpretation of the adjectives included in our scale may be most relevant within the context of the United States. Moreover, we cannot determine whether the relationships observed in predictive validity analyses are causal in nature. Further, the magnitude of some of the validity correlations was modest; additional psychometric research may help to strengthen the case for the construct validity of the POTUS. Nevertheless, the inclusion of high-risk groups—including smokers and the children of smokers—provides a unique opportunity to evaluate prototypes of tobacco users among those who may be most vulnerable to social influences surrounding cigarette smoking and e-cigarette use. Finally, having a similarly worded scale for smoking and vaping may also simplify research among dual users of cigarettes and e-cigarettes. Changes in the prevalence of dual use may have implications for the social images or prototypes that are linked to both. Thus, our scale may be useful in a contemporary setting; however, future work will be needed to determine whether these prototypes persist or whether they should be updated.

## 5. Conclusions

The POTUS is a robust measure of smoker and e-cigarette user prototypes, exhibiting good reliability and construct validity as demonstrated by correlations between prototypes and tobacco-related attitudes, beliefs, and behavior. Accordingly, our scale may be useful to public health researchers seeking to design interventions that reduce tobacco initiation or promote cessation through the manipulation of smoker and e-cigarette user prototypes. The e-cigarette POTUS provides the field with a novel measure of e-cigarette user prototypes. This measure could be particularly useful for tobacco control researchers given the increasing prevalence of e-cigarette use among both youth and adults [18,19,20,21]. Moreover, the POTUS may be applicable to the measurement of prototypes for users of other tobacco products (e.g., cigars, smokeless tobacco) and emerging tobacco delivery systems.

## Figures and Tables

**Table 1:** Prototype adjectives for tobacco users in previous studies.

Prototype Adjectives	Gibbons [12]	Gibbons [10]	Gerrard [13]	Gibbons [1]	Pepper [14]	Blanton [15]	McCool [16]	Gerrard [17]
**Social status**								
Popular				Y		T		C
Cool or sophisticated				Y	Y	T		C
Trashy					Y			
Classy					Y			
**Social interaction**								
Friendly	A	A	A					
Outgoing	A							
Self-assured	A							
Self-confident				Y		T		T
**Intelligence and cognition**								
Smart		A	A	Y		T	C, T	C
Confused				Y		T		
Irrational		A	A					
Intelligent					Y		C, T	
**Physical attractiveness**								
(Un)attractive		A	A	Y	Y	T		
Good-looking								C
Stylish					Y		C, T	
Sexy					Y		C, T	
**Self-focused**								
(In)considerate	A	A	A	Y	Y	T		
Self-centered		A	A	Y	Y	T		
**Motivated**								
Hardworking	A							
Determined	A							
Careless				Y		T		
(In)dependent		A	A	Y	Y	T		T
Reliable		A	A					
Tough					Y		C, T	
Hard							C, T	
**Emotional and physical health**								
Depressed							C, T	
Moody		A	A					
Weak		A	A				C, T	
Bored							C, T	
Stressed							C, T	
Healthy							C, T	
**Other**								
Foolhardy	A							
Dull [boring]				Y		T		C
Immature				Y	Y	T		
Honest		A	A					
Angry							C, T	

A = adult; Y = young adult; T = teenager (adolescent); C = children.

**Table 2:** Prototypes of Tobacco Users Scale (POTUS) correlates.

	Adult Online Study			Adult RCT ^c^		
		Smoker POTUS+	Smoker POTUS–		Smoker POTUS+	Smoker POTUS–
	*n*	*r*	*r*	*n*	*r*	*r*
**Convergent Validity—Cigarette Smoking**						
Cigarette smoking						
Current smoker (%)	1414	0.36 *	−0.42 *			
Frequency (cigarettes/day) ^a^	221	−0.02	−0.06	2121	−0.06 *	−0.02
Quit intentions ^a^	360	−0.17 *	0.16 *	2121	−0.07 *	0.26 *
Cigarette attitudes and beliefs						
Positive subjective norms about quitting smoking				2099	−0.08 *	0.22 *
Worry about consequences of smoking				2003	−0.09 *	0.37 *
Positive pack attitudes				2053	0.14 *	−0.12 *
Negative pack attitudes				1989	−0.09 *	0.35 *
Thinking about harms of smoking ^b^				1876	−0.00	0.20 *
Negative consequences of smoking				2085	−0.19 *	0.41 *
**Predictive Validity (assessed at Week 4 of trial)**						
Forgoing a cigarette in past week				1704	*β* = −0.05 *	*β* = 0.16 *
Cigarette smoking quit attempt in past week				1880	OR = 1.07 ^d^	OR = 1.27 * ^e^
					**E-Cigarette POTUS+**	**E-Cigarette POTUS–**
				***n***	***r***	***r***
**Convergent Validity—E-cigarette Use**						
E-cigarette use						
Current user (*n* (%))				2094	0.13 *	−0.08 *
Frequency (days/week)				525	0.09 *	0.04
E-cigarette social interactions and ads						
Conversation about e-cigarettes in last month				2104	0.10 *	−0.01
Use e-cigarettes because friends or family use them				526	0.08	−0.04
Ever recommended that someone use e-cigarettes				2102	0.19 *	−0.08 *
Saw or heard e-cigarette advertisement in last 30 days				1677	−0.00	−0.03

POTUS = Prototypes of Tobacco Users Scale. For adult RCT, “Saw or heard e-cigarette advertisement in last 30 days” analysis used POTUS+ and POTUS- data from T6; all other analyses used POTUS+ and POTUS- data from T1. ^a^ Analyses restricted to current smokers. ^b^ Semi-partial correlation coefficient adjusted for RCT arm. ^c^ Adult RCT only includes smokers. ^d^ 95% confidence interval [0.95, 1.20]. ^e^ 95% confidence interval [1.15, 1.42]. * *p* < 0.05.

**Table 3:** POTUS correlates with positive and negative attitudes.

	Adult Online Study		Adult RCT, T1		Adult RCT, T6	
**Smoker**	**POTUS+**	**POTUS–**	**POTUS+**	**POTUS–**	**POTUS+**	**POTUS–**
Attitude+	0.39 *	−0.32 *	0.25 *	−0.15 *	0.37 *	−0.14 *
Attitude−	−0.30 *	0.61 *	−0.04	0.38 *	−0.05 *	0.49 *
Attitude Global	0.45 *	−0.60 *	0.22 *	−0.25 *	0.24 *	−0.39 *
**E-Cigarette User**	**POTUS+**	**POTUS–**	**POTUS+**	**POTUS–**	**POTUS+**	**POTUS–**
Attitude+	0.50 *	−0.33 *	0.42 *	−0.21 *	0.44 *	−0.15 *
Attitude–	−0.32 *	0.63 *	−0.14 *	0.38 *	−0.10 *	0.44 *
Attitude Global	0.55 *	−0.55 *	0.37 *	−0.33 *	0.31 *	−0.34 *

POTUS = Prototypes of Tobacco Users Scale. Correlations for smoker and e-cigarette user attitudes were calculated for study samples with complete data for each measure. * *p* < 0.05.

## Supplementary Materials

The following are available online at https://www.mdpi.com/1660-4601/17/17/6081/s1, Table S1: Participant characteristics; Table S2: Survey items for convergent and predictive validity assessment for the smoker and e-cigarette POTUS subscales; Table S3: Factor loadings and psychometric properties of the POTUS; Table S4: Test–retest reliability of smoker POTUS+/– in adult RCT.
