# Supplementary material for: The Prototypes of Tobacco Users Scale (POTUS) for Cigarette Smoking and E-Cigarette Use: Development and Validation
Source: Int J Environ Res Public Health. 2020 Aug 21;17(17):6081. doi: 10.3390/ijerph17176081 (PMC7503746; doi:10.3390/ijerph17176081)
Supplement: Supplementary file 1 [file ijerph-17-06081-s001.pdf]

Table S1. Participant characteristics.

Characteristic	Adult Online Study (*n* = 1,414)		Adult RCT (*n*=2,149)		Adolescent Study (*n*=112)	
	n	%	*n*	%	*n*	%
Age						
18-24 years	350	24.8	323	15.3		
25-39 years	751	53.1	775	36.7		
40-54 years	208	14.7	642	30.4		
55+ years	105	7.4	371	17.6		
Mean (SD)	33.0	(11.5)	39.7	(13.5)	15	(1.5)
Gender[a]						
Female	743	52.5	1,060	48.7	57	50.9
Male	671	47.5	1,039	49.7	54	48.2
Transgender	--	--	34	1.6	1	0.9
Missing	0		16		0	
Sexual Orientation						
Straight	1,254	88.7	1,734	82.5		
Gay, lesbian, or bisexual	139	9.8	368	17.5		
Other	21	1.5	--	--		
Missing	0		47			
Hispanic						
No	1,325	93.7	1,935	91.4	98	87.5
Yes	89	6.3	181	8.6	14	12.5
Missing	0		33		0	
Race						
White	1,143	80.8	751	35.7	23	20.5
Other/multiracial	82	5.9	286	13.6	8	7.1
Black	91	6.5	994	47.3	76	67.9
Asian/Pacific Islander	83	5.9	70	3.3	5	4.5
Missing	15		48		0	
Education (or Parental Education for Adolescents)						
High school graduate or less	150	10.6	677	31.8	33	29.7
Some college	581	41.1	1,021	47.9	59	53.2

College graduate	523	37.0	312	14.6	9	8.1
Graduate degree	160	11.3	121	5.7	10	9.0
Missing	0		18		1	
Low income (≤ 150% of FPL)						
No	--	--	983	45.9	44	39.3
Yes	--	--	1,159	54.1	68	60.7
Missing	--		7		0	
Household income, annual						
$0-$24,999	332	23.5	1,155	54.5		
$25,000-$49,999	471	33.3	538	25.4		
$50,000-$74,999	316	22.4	202	9.5		
$75,000+	294	20.8	224	10.6		
Missing	1		30			
Current Smoker						
No	1,054	74.5	0	0.0	98	87.5
Yes	360	25.5	2,149	100.0	14	12.5
Missing	0		0		0	
Current E-cigarette User						
No	1,214	85.9	1583	74.9	100	89.3
Yes	200	14.1	531	25.1	12	10.7
Missing	0		35		0	

Note. a. Study participants were asked to self-report sex, but not gender, in the Adult Online Study. FPL – Federal Poverty Level.

Table S2. Survey items for convergent and predictive validity assessment for the Smoker and E-cigarette POTUS subscales.

Quit intentions	Adult Online Study	Are you planning to quit smoking…	1=Within the next month<br>2=Within the next 6 months<br>3=Sometime in the future beyond 6 months<br>4=Or are you not planning to quit?	ITC Survey ("International Tobacco Control Policy Evaluation Project,") [36]
Quit intentions	Adult RCT	How interested are you in quitting smoking in the next month?	1=Not at all interested<br>2=A little interested<br>3=Somewhat interested<br>4=Very interested	Adapted from Klein (2009) (Klein, Zajac, & Monin, 2009) [37]
Quit intentions	Adult RCT	How much do you plan to quit smoking in the next month?	1=Not at all<br>2=A little<br>3=Somewhat<br>4=Very much	Adapted from Klein (2009)(Klein, Zajac, & Monin, 2009) [37]
Quit intentions	Adult RCT	How likely are you to quit smoking in the next month?	1=Not at all likely<br>2=A little likely<br>3=Somewhat likely<br>4=Very likely	Adapted from Klein (2009) (Klein, Zajac, & Monin, 2009) [37]
Positive subjective norms about quitting smoking	Adult RCT	People who are important to me think I should quit smoking in the next 2 months.	1=Strongly disagree<br>2=Disagree<br>3=Neither agree nor disagree<br>4=Agree<br>5=Strongly agree	Adapted from Armitage (2007) (Armitage, 2007) [38]
Positive subjective norms about quitting smoking	Adult RCT	People who are important to me would approve of my quitting smoking in the next 2 months.	1=Strongly disagree<br>2=Disagree<br>3=Neither agree nor disagree	Adapted from Armitage (2007) (Armitage, 2007) [38]

			4=Agree<br>5=Strongly agree	
Positive subjective norms about quitting smoking	Adult RCT	People who are important to me want me to quit smoking in the next 2 months.	1=Strongly disagree<br>2=Disagree<br>3=Neither agree nor disagree<br>4=Agree<br>5=Strongly agree	Adapted from Armitage (2007) (Armitage, 2007) [38]
Positive subjective norms about quitting smoking	Adult RCT	When it comes to quitting smoking in the next 2 months, I want to do what people who are important to me think I should do.	1=Strongly disagree<br>2=Disagree<br>3=Neither agree nor disagree<br>4=Agree<br>5=Strongly agree	Adapted from Armitage (2007) (Armitage, 2007) [38]
Worry about consequences	Adult RCT	How worried are you about your health because of your smoking?	1=Not at all worried<br>2=A little worried<br>3=Fairly worried<br>4=Very worried<br>5=Extremely worried	Adapted from Dijkstra (2003) (Dijkstra & Brosschot, 2003) and Ranby (2013) (Ranby et al., 2013) [39,40]
Worry about consequences	Adult RCT	How anxious do you feel when you think of the possible consequences of your smoking?	1=Not at all worried<br>2=A little worried<br>3=Fairly worried<br>4=Very worried<br>5=Extremely worried	Adapted from Dijkstra (2003) (Dijkstra & Brosschot, 2003) and Ranby (2013) (Ranby et al., 2013) [39,40]
Worry about consequences	Adult RCT	How worried are you that your smoking bothers non-smokers?	1=Not at all worried<br>2=A little worried<br>3=Fairly worried<br>4=Very worried<br>5=Extremely worried	Adapted from Magnan (2009) (Magnan et al., 2009) and Magnan (2013) (Magnan et al., 2013) [39,40]
Worry about consequences	Adult RCT	How worried are you that your smoking can be harmful to other people?	1=Not at all worried<br>2=A little worried<br>3=Fairly worried<br>4=Very worried<br>5=Extremely worried	Adapted from Magnan (2009) (Magnan et al., 2009) and Magnan (2013) (Magnan et al., 2013) [39,40]

Positive pack attitudes	Adult RCT	Thinking about the cigarette pack you're using now, do you think the pack looks…?	1=Very uncool<br>2=Somewhat uncool<br>3=Somewhat cool<br>4=Very cool	Adapted from Moodie et al. (2011) (Moodie et al., 2011) [41]
Positive pack attitudes	Adult RCT	Thinking about the cigarette pack you're using now, do you think the pack looks…?	1=Very unattractive<br>2=Somewhat unattractive<br>3=Somewhat attractive<br>4=Very attractive	Adapted from Moodie et al. (2011) (Moodie et al., 2011) [41]
Positive pack attitudes	Adult RCT	Thinking about the cigarette pack you're using now, do you think the pack looks…?	1=Very unappealing<br>2=Somewhat unappealing<br>3=Somewhat appealing<br>4=Very appealing	Adapted from Moodie et al. (2011) (Moodie et al., 2011) [41]
Negative pack attitudes	Adult RCT	Thinking about the cigarette pack you're using now, how much does the pack make you feel…Embarrassed?	1=Not at all<br>2=A little<br>3=Somewhat<br>4=Very<br>5=Extremely	Adapted from Moodie et al. (2011) (Moodie et al., 2011) [41]
Negative pack attitudes	Adult RCT	Thinking about the cigarette pack you're using now, how much does the pack make you feel…Ashamed?	1=Not at all<br>2=A little<br>3=Somewhat<br>4=Very<br>5=Extremely	Adapted from Moodie et al. (2011) (Moodie et al., 2011) [41]
Negative pack attitudes	Adult RCT	Thinking about the cigarette pack you're using now, how much does the pack make you feel…Unaccepted?	1=Not at all<br>2=A little<br>3=Somewhat<br>4=Very<br>5=Extremely	Adapted from Moodie et al. (2011) (Moodie et al., 2011) [41]
Thinking about harms of smoking	Adult RCT	In the last week, how often did you…<br>Think about how much you enjoy smoking?	1=Never<br>2=Rarely<br>3=Sometimes<br>4=Often<br>5=All of the time	Adapted from Borland et al. (2009) [42]

Thinking about harms of smoking	Adult RCT	Think about the harm your smoking might be doing to you?	1=Never<br>2=Rarely<br>3=Sometimes<br>4=Often<br>5=All of the time	Adapted from Borland et al. (2009) [42]
Thinking about harms of smoking	Adult RCT	Think about the harm your smoking might be doing to other people?	1=Never<br>2=Rarely<br>3=Sometimes<br>4=Often<br>5=All of the time	Adapted from Borland et al. (2009) [42]
Negative consequences of smoking	Adult RCT	I'm embarrassed that I have to smoke.	1=Strongly disagree<br>2=Disagree<br>3=Neither agree nor disagree<br>4=Agree<br>5=Strongly agree	Adapted from Velicer et al. (1985) (Velicer et al., 1985) [42]
Negative consequences of smoking	Adult RCT	My cigarette smoking bothers other people.	1=Strongly disagree<br>2=Disagree<br>3=Neither agree nor disagree<br>4=Agree<br>5=Strongly agree	Adapted from Velicer et al. (1985) (Velicer et al., 1985) [42]
Negative consequences of smoking	Adult RCT	People think I'm foolish for smoking.	1=Strongly disagree<br>2=Disagree<br>3=Neither agree nor disagree<br>4=Agree<br>5=Strongly agree	Adapted from Velicer et al. (1985) (Velicer et al., 1985) [42]
Negative consequences of smoking	Adult RCT	Smoking makes me seem less attractive.	1=Strongly disagree<br>2=Disagree<br>3=Neither agree nor disagree<br>4=Agree<br>5=Strongly agree	Adapted from Brandon & Baker, 1991 [42]
Conversation about e-cigarettes in last month	Adult RCT	In the last month, how many people did you talk to about e-cigarettes?	1=No one<br>2=1-2 people	Hall et al. (2016) (Hall et al., 2016) [43]

			3=3-4 people<br>4=5 or more people	
Use e-cigarettes because friends or family use them	Adult<br>RCT	Why do you use e-cigarettes or other vaping devices?-My friends or family use them	0=No<br>1=Yes	Hall et al. (2016) (Hall et al., 2016) [43]
Ever recommended that someone use e-cigarettes	Adult<br>RCT	Have you ever recommended that someone use e-cigarettes?	0=No<br>1=Yes	Hall et al. (2016) (Hall et al., 2016) [43]
Saw or heard e-cigarette advertisement in last 30 days	Adult<br>RCT	In the last 30 days, have you seen or heard any advertisements for e-cigarettes?	0=No<br>1=Yes	--
Forgoing a cigarette	Adult<br>RCT	In the last week, how often have you stopped yourself from having a cigarette because you wanted to <u>smoke less</u>?	1=Never<br>2=1-2 times<br>3=3-4 times<br>4=5-9 times<br>5=10 or more times	Adapted from Borland & Hill (1997) (Borland & Hill, 1997) and Li et al. (2015) (Li et al., 2015) [44, 45]
Forgoing a cigarette	Adult<br>RCT	In the last week, how often have you butted out a cigarette before you finished it because you wanted to <u>smoke less</u>?	1=Never<br>2=1-2 times<br>3=3-4 times<br>4=5-9 times<br>5=10 or more times	Adapted from Li et al. (2015) (Li et al., 2015) [45]
Quit attempts	Adult<br>RCT	In the last <u>week</u>, did you stop smoking for 1 day or longer because you were trying to quit smoking?	1=Yes<br>2=No	Adapted from Centers for Disease Control and Prevention (2010) [46]

-- = developed by authors

Reference

36. Fong, G.T.; Cummings, K.M.; Borland, R.; Hastings, G.; Hyland, A.; Giovino, G.A.; Hammond, D.; Thompson, M.E. The conceptual framework of the International Tobacco Control (ITC) Policy Evaluation Project. *Tob. Control* **2006**, *15* (Suppl 3), iii3–iii11.
37. Klein, W.M.; Zajac, L.E.; Monin, M.M. Worry as a moderator of the association between risk perceptions and quitting intentions in young adult and adult smokers. *Ann. Behav. Med.* **2009**, *38*, 256–261.
38. Armitage, C.J. Efficacy of a brief worksite intervention to reduce smoking: The roles of behavioral and implementation intentions. *J. Occup. Health Psychol.* **2007**, *12*, 376–390.
39. Dijkstra, A.; Brosschot, J. Worry about health in smoking behaviour change. *Behav. Res. Ther.* **2003**, *41*, 1081–1092.
40. Ranby, K.W.; Lewis, M.A.; Toll, B.A.; Rohrbaugh, M.J.; Lipkus, I.M. Perceptions of smoking-related risk and worry among dual-smoker couples. *Nicotine Tob. Res.* **2013**, *15*, 734–738.
41. Moodie, C.; Mackintosh, A.M.; Hastings, G.; Ford, A. Young adult smokers' perceptions of plain packaging: A pilot naturalistic study. *Tob. Control* **2011**, *20*, 367–373.
42. Fathelrahman, A.I.; Omar, M.; Awang, R.; Cummings, K.M.; Borland, R.; Samin, A.S.B.M. Impact of the new Malaysian cigarette pack warnings on smokers' awareness of health risks and interest in quitting smoking. *Int. J. Environ. Res. Public Health* **2010**, *7*, 4089–4099.
43. Hall, M.G.; Pepper, J.K.; Morgan, J.C.; Brewer, N.T. Social interactions as a source of information about E-cigarettes: A study of U.S. adult smokers. *Int. J. Environ. Res. Public Health* **2016**, *13*, 788.
44. Borland, R.; Hill, D. Initial impact of the new Australian tobacco health warnings on knowledge and beliefs. *Tob. Control* **1997**, *6*, 317–325.
45. Li, L.; Borland, R.; Fong, G.T.; Jiang, Y.; Yang, Y.; Wang, L.; Thrasher, J.F. Smoking-related thoughts and microbehaviours, and their predictive power for quitting. *Tob. Control* **2015**, *24*, 354–361, doi:10.1136/tobaccocontrol-2013-051384
46. McClave, A.K.; Whitney, N.; Thorne, S.L.; Mariolis, P.; Dube, S.R.; Engstrom, M. Adult tobacco survey—19 States, 2003–2007. *MMWR Surveill. Summ.* **2010**, *59*, 1–75.

Table S3. Factor loadings and psychometric properties of the POTUS.

	Adjectives	**Adult Online Study** (*n*=1,414)		**Adult RCT T1** (*n*=2,145)		**Adult RCT T6** (*n*=1,895)		**Adolescent Study** (*n*=112)	
**Smoker**									
POTUS+	Cool	0.50		0.59		0.70		0.48	
POTUS+	Sexy	0.61		0.64		0.72		0.58	
POTUS+	Smart	0.79		0.86		0.90		0.39	
POTUS+	Healthy	0.76		0.59		0.74		0.54	
POTUS–	Disgusting		0.84		0.81		0.86		0.63
POTUS–	Unattractive		0.77		0.72		0.83		0.66
POTUS–	Immature		0.69		0.68		0.81		0.61
POTUS–	Inconsiderate		0.74		0.72		0.82		0.62
r between POTUS+ and POTUS-		-.48		.11		.20		0.27	
	Cronbach's alpha	0.80	0.85	0.79	0.82	0.87	0.90	0.54	0.73
	CFI	0.98		0.98		0.99		1.00	
	RMSEA	0.06		0.06		0.06		0.01	
	**Adjectives**	**Adult Online Study** (*n*=1,414)		**Adult RCT T1** (*n*=2,142)		**Adult RCT T6** (*n*=1,888)		**Adolescent Study** (*n*=112)	
**E-cigarette user**									
POTUS+	Cool	0.63		0.73		0.77		0.42	
POTUS+	Sexy	0.60		0.66		0.72		0.55	
POTUS+	Smart	0.83		0.83		0.87		0.66	
POTUS+	Healthy	0.82		0.82		0.84		0.58	
POTUS–	Disgusting		0.85		0.82		0.87		0.70
POTUS–	Unattractive		0.82		0.78		0.83		0.61
POTUS–	Immature		0.78		0.78		0.84		0.69
POTUS–	Inconsiderate		0.76		0.76		0.84		0.52
r between POTUS+ and POTUS-		-.43		.13		.20		-0.25	
	Cronbach's alpha	0.84	0.88	0.86	0.86	0.89	0.91	0.65	0.72
	CFI	0.98		0.99		0.99		1.00	
	RMSEA	0.07		0.06		0.07		0.00	

Note. CFI – Comparative Fit Index, RMSEA – Root Mean Square Error of Approximation.

Table S4. Test-retest reliability of smoker POTUS+/– in Adult RCT.

	**T1**	**T2**	**T3**	**T4**	**T5**	**T6**
T1		0.67*	0.58*	0.61*	0.56*	0.54*
T2	0.66*		0.62*	0.64*	0.62*	0.59*
T3	0.52*	0.58*		0.77*	0.67*	0.65*
T4	0.54*	0.62*	0.63*		0.77*	0.71*
T5	0.50*	0.58*	0.59*	0.71*		0.74*
T6	0.46*	0.51*	0.59*	0.64*	0.66*	

Note. Correlations were calculated for Adult RCT samples with complete data at each time point. Smoker POTUS+ correlations below diagonal. Smoker POTUS– correlations above diagonal. *$p < 0.05$.
